# Microfluidic time‐lapse analysis and reevaluation of the *Bacillus subtilis* cell cycle

**DOI:** 10.1002/mbo3.876

**Published:** 2019-06-13

**Authors:** Seoungjun Lee, Ling Juan Wu, Jeff Errington

**Affiliations:** ^1^ Centre for Bacterial Cell Biology, Institute for Cell and Molecular Biosciences, Medical School Newcastle University Newcastle‐upon‐Tyne UK; ^2^ Present address: Department of Basic and Clinical Neuroscience, Maurice Wohl Clinical Neuroscience Institute, Institute of Psychiatry, Psychology and Neuroscience King’s College London London UK

**Keywords:** adder, cell size homeostasis, single‐cell analysis, sizer

## Abstract

Recent studies taking advantage of automated single‐cell time‐lapse analysis have reignited interest in the bacterial cell cycle. Several studies have highlighted alternative models, such as Sizer and Adder, which differ essentially in relation to whether cells can measure their present size or their amount of growth since birth. Most of the recent work has been done with *Escherichia coli*. We set out to study the well‐characterized Gram‐positive bacterium, *Bacillus subtilis,* at the single‐cell level, using an accurate fluorescent marker for division as well as a marker for completion of chromosome replication. Our results are consistent with the Adder model in both fast and slow growth conditions tested, and with Sizer but only at the slower growth rate. We also find that cell size variation arises not only from the expected variation in size at division but also that division site offset from mid‐cell contributes to a significant degree. Finally, although traditional cell cycle models imply a strong connection between the termination of a round of replication and subsequent division, we find that at the single‐cell level these events are largely disconnected.

## Graphical abstract

We have studied the *Bacillus subtilis* cell cycle at two different growth rates, using microfluidic system and fluorescent markers for the cell membrane and chromosomal terminus. The results fit with an Adder‐type model at both growth rates, and with Sizer at the slower growth rates. We find, unexpectedly, that division timing is uncoupled from the termination of chromosome replication.

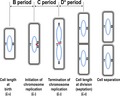

## 1 INTRODUCTION

**Figure 1:**
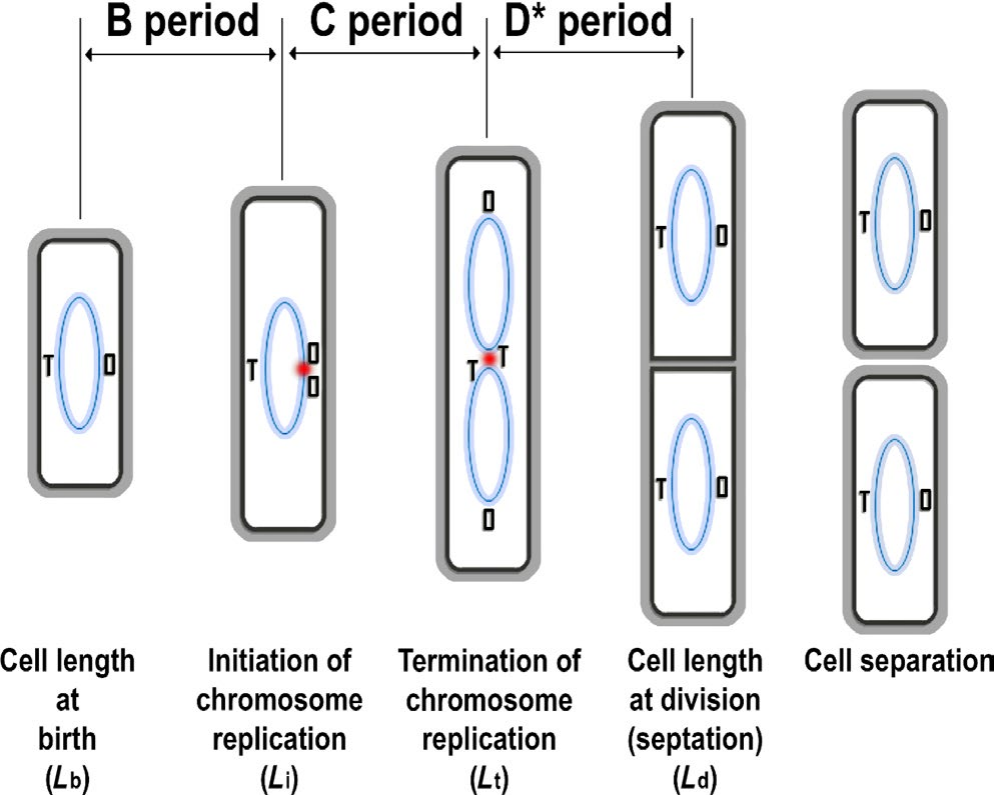
Schematic view of the bacterial cell cycle. Blue ovals represent chromosomes. O and T represent, respectively, the origin and terminus sites for chromosome replication. The red dot indicates initiation or termination events. Note that in many bacteria growing rapidly, rounds of DNA replication can overlap, creating more complicated cell cycle patterns. Unlike Gram‐negative bacteria, in which constriction at the division site and separation of sister cells occur more or less simultaneously, in Gram‐positive bacteria, cells can remain connected together via common wall material in the division septum for a protracted and relatively variable period of time. We therefore previously defined the completion of septation in *Bacillus subtilis* as equivalent to division in *Escherichia coli*, and defined the period between completion of replication and septation as D* (Sharpe et al., 1998)

The cell division cycle is one of the most extensively studied processes in biology. In bacteria, the classic view was established in the 1950s and 1960s, based largely on studies of *Escherichia coli* (Cooper & Helmstetter, 1968; Donachie, 1968; Kubitschek, 1966, 1968, 1969; Perry, 1959) but thought generally to be similar in other symmetrically dividing rod‐shaped bacteria (e.g., *Bacillus subtilis*; Sharpe, Hauser, Sharpe, & Errington, 1998). The scheme is summarized briefly in Figure 1. In rod‐shaped organisms cell length is roughly proportional to cell mass or volume and because length is convenient to measure (Donachie, 1968; Grover, Woldringh, Zaritsky, & Rosenberger, 1977), critical moments in the cell cycle tend to be defined based on this (e.g., Sharpe et al., 1998; Taheri‐Araghi et al., 2015; Sauls, Li, & Jun, 2016; Zheng et al., 2016). For any given growth condition, the newborn cell has a length *L*_b_. The cell grows exponentially at a rate proportional to its length. At a critical length *L*_i_, corresponding to the “initiation mass” (Donachie, 1968), the cell initiates a round of chromosome replication. After a fixed period of time (the C period), which was thought largely to be independent of growth rate, the round of chromosome replication terminates, at which time the cell has reached a length *L*_t_. Cell division follows, a fixed period of time later (called the D period; or D* – see below) at a length *L*_d_ *,* approximately twice that of *L*_b_. This model, often called the Cooper–Helmstetter model (Cooper & Helmstetter, 1968), satisfied many observed features of the *E. coli* cell cycle, particularly changes in average cell size according to growth rate (faster growing cells tend to be larger than slow growing cells) (Cullum & Vicente, 1978). Its central assumptions included the ability of the cell to sense the initiation mass and dependence of division timing on constancy of the C and D periods. Note that, although Figure 1 shows a simple cell cycle representative of slow growing cells, at faster growth rates, initiation of chromosome replication occurs prior to the previous cell division, so that fast growing cells can contain multiple chromosome origins.

The early experiments providing the evidence that led to the Cooper–Helmstetter model were mainly based on population studies, in which the average behavior of cells was measured (Cooper & Helmstetter, 1968). In the last few years, the advent of accurate, automated time‐lapse microscopy has enabled the model to be tested at the level of individual cell behavior (Campos et al., 2014; Potvin‐Trottier, Luro, & Paulsson, 2018; Sauls et al., 2016; Taheri‐Araghi et al., 2015; Wallden, Fange, Lundius, Baltekin, & Elf, 2016; Yu et al., 2017; Zheng et al., 2016). Surprisingly, these experiments have led to a reevaluation of cell cycle behavior. The classical model falls into a class of models sometimes called “Sizer,” in which the cell can sense its mass and use this information to control cell cycle events. An interesting alternative to this model, which has received considerable attention recently, is sometimes called “Incremental” or “Adder” and is based on the notion that the cell is oblivious to its starting mass but instead divides after the addition of a constant amount of new material (Amir, 2014; Campos et al., 2014; Fantes & Nurse, 1981; Modi, Vargas‐Garcia, Ghusinga, & Singh, 2017; Sauls et al., 2016). The two models both assume that the cell can somehow “measure” mass – Sizer measures the mass appropriate for division and Adder the amount of new growth. Both models can account for population‐based measures of cell cycle progression. A third model called Timer, assumes that cells grow for a relatively fixed period of time between divisions but this is not particularly effective at supporting size homeostasis.

Unfortunately, there is not yet a clear consensus on the extent to which pure Sizer or Adder models are appropriate descriptions of the cell cycle, less so, the molecular mechanisms that could underpin these phenomena. Also, most work has been done on the Gram‐negative bacteria, *E. coli* (Bertaux, Marguerat, & Shahrezaei, 2018; Campos et al., 2014; Furse, Wienk, Boelens, Kroon, & Killian, 2015; Hill, Kadoya, Chattoraj, & Levin, 2012; Osella, Nugent, & Lagomarsino, 2014; Wallden et al., 2016; Zheng et al., 2016) and *Caulobacter crescentus* (Banerjee et al., 2017; Campos et al., 2014; Woldemeskel & Goley, 2017; Wright et al., 2015). The cell cycle of the Gram‐positive bacterium, *B. subtilis*, has been studied in considerable detail previously but mainly at the population level (Burdett, Kirkwood, & Whalley, 1986; Holmes, Rickert, & Pierucci, 1980; Nanninga, Koppes, & Vries‐Tijssen, 1979; Paulton, 1971; Sargent, 1975; Sharpe et al., 1998). Two clear differences from *E. coli* are apparent. First, unlike *E. coli*, which varies in both cell length and width according to the growth rate, *B. subtilis* changes only its length (Sharpe et al., 1998; Weart et al., 2007). Second, in *B. subtilis,* the processes of septation (membrane scission) and cell separation (wall scission) are temporally disconnected, whereas in *E. coli* they occur simultaneously (Errington, Daniel, & Scheffers, 2003). As the cell separation time is quite variable in *B. subtilis*, depending both on growth conditions and cell to cell differences (Holmes et al., 1980; Nanninga et al., 1979), we previously defined a D* period, corresponding to the interval between termination of replication and membrane scission, which is relatively constant when measured at the population average level (Sharpe et al., 1998). Moreover, because the FtsZ‐based division machine, which is almost universal in bacteria, operates during membrane scission rather than cell separation, D* is probably functionally equivalent to the D period of *E. coli* (Errington et al., 2003; Harry, 2001). The only report of time‐lapse analysis on individual growing cells of *B. subtilis*, was based on phase contrast imaging (Taheri‐Araghi et al., 2015), which detects cell separation rather than scission, and which we show are temporally separated events.

Here, we take advantage of an automated system for measurement of the growth and division of *B. subtilis* cells, over many generations, in an agarose‐based microfluidic device (Eland, Wipat, Lee, Park, & Wu, 2016; Moffitt, Lee, & Cluzel, 2012). We have also developed fluorescent tools for measuring DNA replication and particularly the membrane steps of the cell cycle. We find that for two growth media, conferring different growth rates, the *B. subtilis* cycle tends to follow an Adder‐like model, but that the accuracy of cell size homeostasis depends on the growth rate. We also report an unexpected contribution to cell birth size variation through division asymmetry, despite the common assumption that *E. coli* and vegetative *B. subtilis* cells divide with considerable precision at mid‐cell. Finally, we have tested the dependence of cell division on the chromosome cycle and found, surprisingly, that in individual cells there is little or no apparent coupling between the termination of chromosome replication and cell division, so the nucleoid is unlikely to provide the cell mass or length “ruler” needed for an Adder model.

## 2 EXPERIMENTAL PROCEDURES

### 2.1 Strains

Strains and plasmids used are listed in Table A2.

The WALP23 artificial transmembrane helix (sequence AW_2_L(AL)_8_W_2_A) was previously shown, when fused to mGFP, to provide a good general membrane label when expressed in *B. subtilis* (Scheinpflug et al., 2017). The Scheinpflug construct was under xylose‐inducible control. To be able to express the fusion constitutively, we removed part of the xylose operator sequence from the original construct. First, nine nucleotides in the middle of the operator sequence (TTTGGGCAA) in plasmid pL015 were removed by site‐directed mutagenesis (SDM) using primers 5’‐GATTAAAATAAGTTAGTTTGCAAACTAATGTGCAACTTACTTAC‐3’ and 5’‐GTAAGTAAGTTGCACATTAGTTTGCAAACTAACTTATTTTAATC‐3’. The resulting plasmid (pL062) was transformed into the *B. subtilis* wild‐type strain 168CA, resulting in strain sL105. sL105 was subsequently transformed with plasmids pL006 (*tetR‐mCherry*) and pCRW10 (*dacC::tetO* at 171°, termination site) to generate strain sL099.

### 2.2 Growth media

Strains were grown in either SM (slow‐growth medium) or FM (fast‐growth medium). SM contained 0.002 mg/ml C_6_H8FeNO_7_, 0.200 mg/ml tryptophan, 40 mg/ml glucose, 10 mg/ml L‐glutamate, 2 mg/ml NaCl, 1 mg/ml yeast extract, 2 mg/ml tryptone, 0.14% (w/v) H_8_N_2_O_4_S, 0.98% (w/v) HK_2_O_4_P, 0.42% (w/v) H_2_KO_4_P, 0.07% (w/v) C_6_H_9_Na_3_O_9_ and 0.014% (w/v) MgO_4_S. FM contained 0.02 mg/ml tryptophan, 1.20 mg/ml MgSO_4_, 3.90 mg/ml glucose, 976 mg/ml NaCl, 488 mg/ml yeast extract, 976 mg/ml tryptone, 0.176% (w/v) H_8_N_2_O_4_S, 1.23% (w/v) HK_2_O_4_P, 0.53% (w/v) H_2_KO_4_P, 0.088% (w/v) C_6_H_9_Na_3_O_9,_ and 0.018% (w/v) MgO_4_S.

### 2.3 Microscopy

Microscopy experiments were performed on an inverted fluorescence microscope (Nikon Eclipse Ti body with Perfect Focus) with oil immersion objectives (either an Apo TIRF Nikon 60×/1.49 or Plan Apo Nikon 60×/1.4). A 300 Watt xenon arc lamp equipped with a liquid light guide (Sutter Instruments, Lambda 10–3) and a Prime sCMOS camera (Photometrics) were used for florescent, phase‐contrast and bright field imaging. The illumination light was always used with a UV filter, and the exposure time of bright field, GFP, and mCherry was 100 ms, 200 ms, and 200 ms, respectively. For microfluidic microscopy, images were captured at intervals of 2 min for up to 11 hr when cells were grown in SM at 32°C, and 1 min for up to 8 hr in the FM at 32°C. The Perfect Focus System (Nikon) was used in all experiments.

### 2.4 Agarose‐based microfluidics

An agarose‐based microfluidic system, consisted of a PDMS chamber that housed an agarose pad, a syringe pump and a microscope (Eland et al., 2016; Moffitt et al., 2012) was used. Cells were cultured overnight in FM or SM then diluted back (1:1,000 dilution) and regrown for 2–4 hr to early exponential phase. A sample was transferred to the prewarmed microfluidic device and grown in agarose channels in a PDMS chamber. The microfluidic device was homemade. Briefly, a PDMS block was bonded to a cover glass (Agar Scientific, Coverglass 35x64 mm No.1.5) using a plasma cleaner (Harrick Plasma) to create a chamber. The glass bottom of the chamber was coated with bovine serum albumin (BSA, 50 mg/ml) and left to dry for 30 min to prevent cells from sticking to the glass surfaces. Two microliters of cells were added to the glass bottom of the microfluidic chamber, then an agarose pad was placed on the culture to trap cells in the channels on the agarose pad. The agarose pad, prepared using 5% low melting point agarose (Lonsza, SeaPlaque GTG Agarose) containing growth medium, had channels of three different widths (0.8 µm, 0.9 µm, and 1.0 µm wide). Subsequently, the top of the PDMS chamber was sealed by bonding to a plasma‐cleaned plastic cover slip (Agar Scientific, plastic cover slips 22 × 22 mm). Then, through microbore tubing (Tygon, 0.020" × 0.060"OD), one of the two buffer reservoirs in the chamber was connected to a 50‐ml syringe that contained the growth medium and was controlled by a syringe pump (New Era Pump Systems Inc, NE‐1000), and the other to a flask to collect spent medium and overgrown cells. The microscope was enclosed in an incubator chamber (Solent Scientific) maintained at a constant temperature of 32°C (by allowing equilibration to temperature for at least 5 hr before the microfluidic device was introduced into the chamber). Cells grew along straight microfluidic channels molded into the agarose pad (Supporting Information Movies S1, S2 and S3). Time‐lapse imaging was initiated after 30 min to allow cells to acclimatize to the growth conditions.

### 2.5 Image processing and analysis

**Figure A1:**
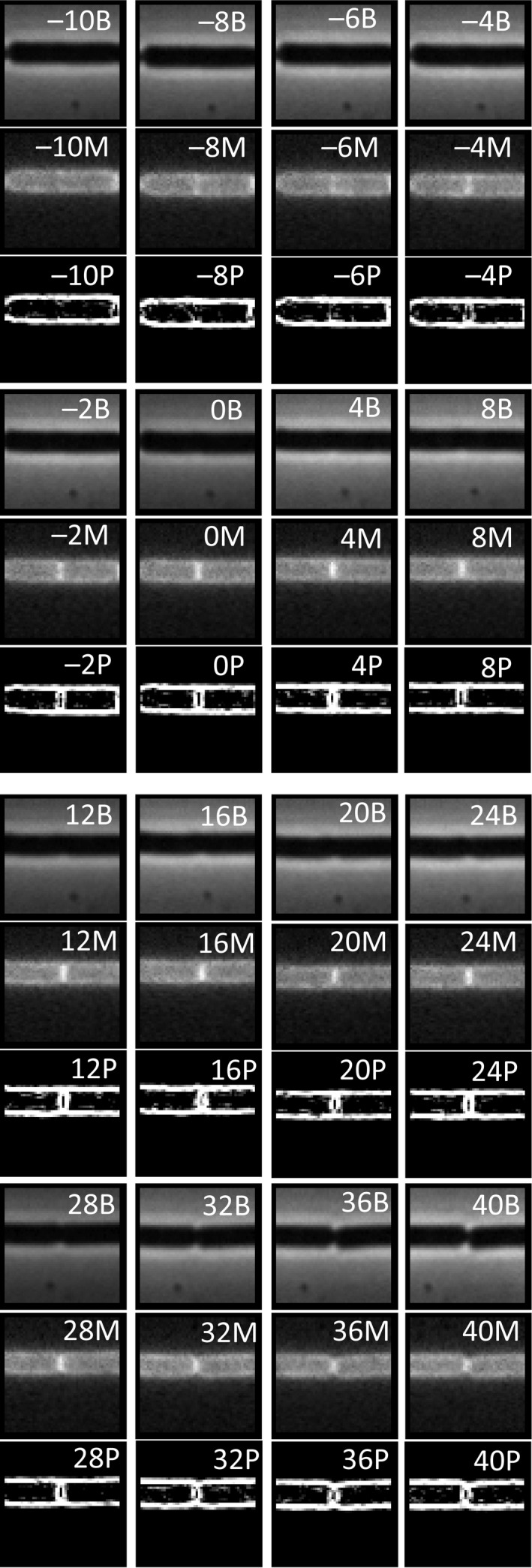
Detection of cell septation by fluorescence and comparison with bright field images. Bright field images (B), fluorescent membrane protein images (M) and post‐processed fluorescence images (P) of strain sL105 in a microfluidic channel. Images are in time order, with 2 minutes between frames. The septation event visible in the fluorescence image and defined by the clear, closed, membrane ring in the post‐processed image defines time zero. Other frames are arranged in sequence with times indicated relative to that of the zero frame (min). Note that division was undetectable by bright field microscopy until about + 12 or + 16 min [Correction added on 5 July 2019 after first online publication: Figure A1 has been corrected]

Cell membrane and termination data from the microfluidic experiments were image‐processed using FIJI‐ImageJ (Schindelin et al., 2012). Images of cell membrane fluorescent tag were first processed to remove background signal, adjust contrast and brightness, and to apply edge processing. After that, the membrane signal was selected as a bold line, to provide a clear cell boundary. Following image processing, even unclear division septa were well defined (see Appendix Figure A1). Images of the replication termination fluorescent tag were also processed to remove background signal, adjust contrast and brightness, and apply gamma processing. Following processing, the termination spots could be seen clearly without blurred boundaries (Figure 6a4).

Cell division time was recorded as the time difference between birth of mother and daughter cells. Cell division (membrane scission) was verified by the appearance of a clear, intact membrane ring at mid‐cell (“0P” in Appendix Figure A1). Cell birth length and termination length were measured using MicrobeTracker (Sliusarenko, Heinritz, Emonet, & Jacobs‐Wagner, 2011) and verified, where necessary, by visual inspection. Because termini could move in three dimensions, two termini separated in the Z plane could be mistaken for an unreplicated terminus. We interpreted elongated or unusually bright foci in frames just preceding those with two clearly separated foci as containing two overlapping terminus foci, to partially compensate for delayed recognition of separation.

### 2.6 Data processing

With the microfluidic images, we measured the cells collected over a series of generations to give a continuous cell pedigree. Recordings covered 6 generations for FM and 11 generations for SM. During the time‐lapse microscopy, we excluded from analysis: (a) cells that had more than two termination spots, (b) cells that were extremely long at birth, or not dividing, or having multiple divisions, (c) cells at the exit of the channel in the agarose pad, and (d) cells growing side by side with another chain of cells in the same channel. These criteria were applied to all of the single‐cell data obtained with both SM and FM.

All of the single‐cell data, including birth length, termination time, and termination length, were validated by visual inspection of the cell boundaries or spots. All data were processed in MATLAB (MathWorks). The MATLAB code was custom‐made to support automated analysis for the time‐lapse imaging data.

In fitting a linear trend line to the single‐cell data, up to 5% of the data, including 2.5% of the minimum values and 2.5% of the maximum values, were excluded. This helped to avoid systematic errors in the range of low sample numbers. The mean absolute error (MAE) was used to provide a goodness‐of‐fit measure between linear trend lines and experimental data.

## 3 RESULTS AND DISCUSSION

### 3.1 Long‐term cell cycle analysis by time‐lapse fluorescence imaging

As phase contrast microscopy does not detect septum formation with precision in *B. subtilis* (see above), we tested a range of fluorescence‐based methods for ability to detect septal closure while minimizing phototoxicity and bleaching, to enable long‐term time‐lapse imaging. We eventually fixed on a constitutively expressed monomeric GFP (mGFP) fusion to an artificial model transmembrane helix, WALP23‐mGFP (Scheinpflug et al., 2017; see Experimental Procedures), which clearly labeled membranes and showed the progression of septation. A UV filter was used to reduce DNA damage, and a highly sensitive objective lens (TIRF ×60, NA1.49), which was approximately 30% more sensitive was used instead of the standard 100× lens. In some experiments, we also used a TetR‐mCherry fusion targeted to the extreme terminus region of the chromosome, so that we could use focus duplication to time the completion of chromosome replication relative to cell division events. We also developed an automated system for tracking growth and division of individual cells through multiple generations. Two low background fluorescence media recipes were developed giving different growth rates, “Fast Medium” (FM) and “Slow Medium” (SM), while minimizing image capture times and thus phototoxic effects (Supporting Information S1, S2 and S3). In control experiments using bright field imaging only (i.e., no fluorescence irradiation) the measured growth rates were indistinguishable from those of the irradiated cultures. Appendix Figure A1 shows that septation was detected by fluorescence and image processing several frames before it became visible by bright‐field microscopy.

### 3.2 Steady‐state growth under microfluidic conditions

**Figure 2:**
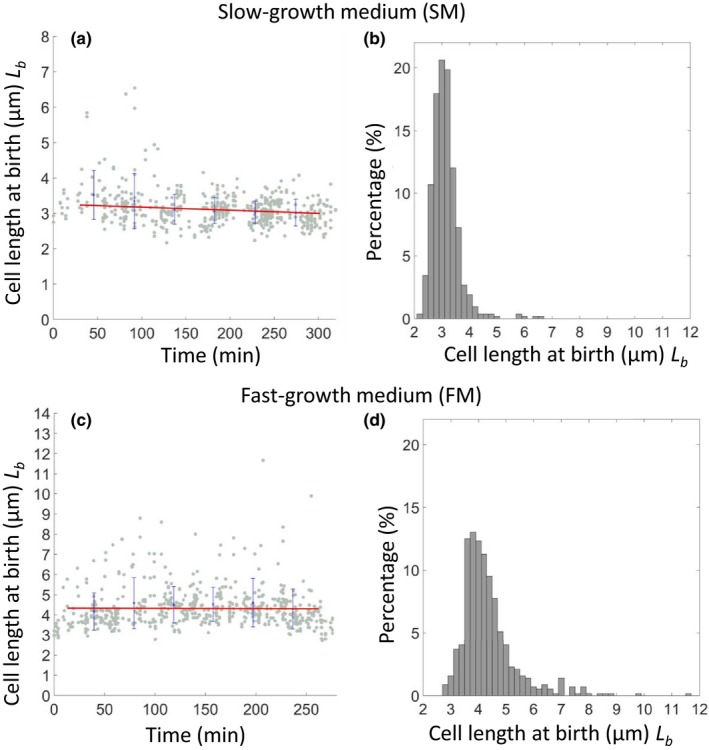
Stable growth of *Bacillus subtilis* in agarose‐based microfluidics. A derivative of the wild type *B. subtilis* strain (sL105) was grown in SM (a, b) and FM (c, d) in agarose‐based microfluidic channels at 32°C. Strains were recorded in SM for about 6 hr through 6 generations (*n* = 524) and in FM for about 6 hr through 11 generations (*n* = 568). (a, c) show plots of cell length at birth against elapsed time. Gray dots show each single‐cell data point, and blue crosses and lines show the average of binned data and errors (standard deviation). (b, d) show histograms of cell length distribution at birth

To check that the microfluidic conditions could support long‐term steady‐state growth of *B. subtilis* we grew the cells in FM or SM at 32°C and measured various cell cycle parameters over a prolonged time period (320 min in SM and 280 in FM), during which up to 6 (SM) and 11 (FM) sequential cell division events were captured (Appendix Table A1). Figure 2a,c shows that steady‐state growth over many hours was achieved under both media conditions. The average interdivision (i.e., generation or doubling) times were 25 ± 5 min for FM and 57 ± 11 min for SM. Average lengths at birth (<*L*_b_>) were 4.36 ± 1.02 µm and 3.13 ± 0.50 µm, respectively. Figure 2a,c also shows how these values varied during a long (6 hr) time‐lapse experiment. Apart from a slight reduction in cell size toward the end of the experiment, especially in FM, growth parameters were well maintained.

### 3.3 Cell size homeostasis and the Adder model

Recent work using time‐lapse imaging, mainly focused on *E. coli*, has prompted a paradigmatic shift in our view of bacterial cell cycle control, with the finding that cells can achieve size homeostasis via a growth increment or “Adder” mechanism (Campos et al., 2014; Eun et al., 2018; Taheri‐Araghi et al., 2015). *B. subtilis* is very far from *E. coli* in terms of evolution (Gram‐positive vs. Gram‐negative), yet data from Taheri‐Araghi et al. (2015) suggested that it too uses an Adder mechanism. We noticed that Taheri‐Araghi et al. (2015) used phase‐contrast microscopy to measure the time of division, so it seemed useful to examine whether our WALP23‐mGFP construct, providing improved temporal precision on the time of division, would concur with those earlier results.

**Figure A2:**
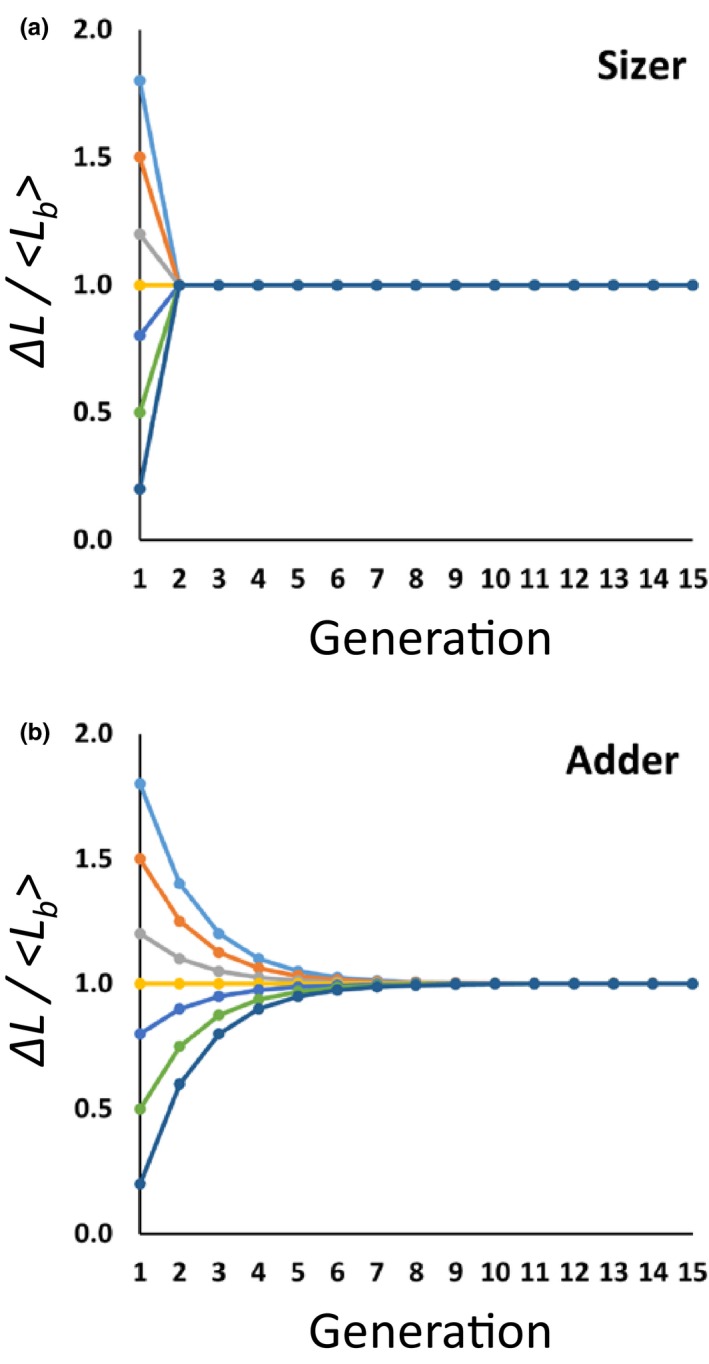
Patterns of cell size convergence to the mean for Adder and Sizer homeostasis models. (A) Simulation of cell size convergence based on simulation data for the Sizer model (B) Equivalent data for the Adder model [Correction added on 5 July 2019 after first online publication: Figure A2 has been corrected]

Sizer and Adder models make different predictions about the relationship between cell length at birth (*L*_b_) and subsequent division (*L*_d_). Sizer assumes that the cell can sense its size or mass and divides when it reaches a certain size: in other words, it can regulate the timing of division to compensate for variations in size at birth. This, in principle, results in correction of variation from the average *L*_b_, immediately, at the next division (Appendix Figure A2a). In contrast, Adder is oblivious to size at birth or division and works by allowing a fixed increment of growth, corresponding to the preferred (average) population size, before next division. This model predicts that size correction will occur over several generations, with a recurring “memory” of previous cell size (Appendix Figure A2b). In practice, both models need to be adjusted to take account of an inevitable level of stochastic variation in the precision of the timing and positioning (central or off‐central) of division.

**Figure 3:**
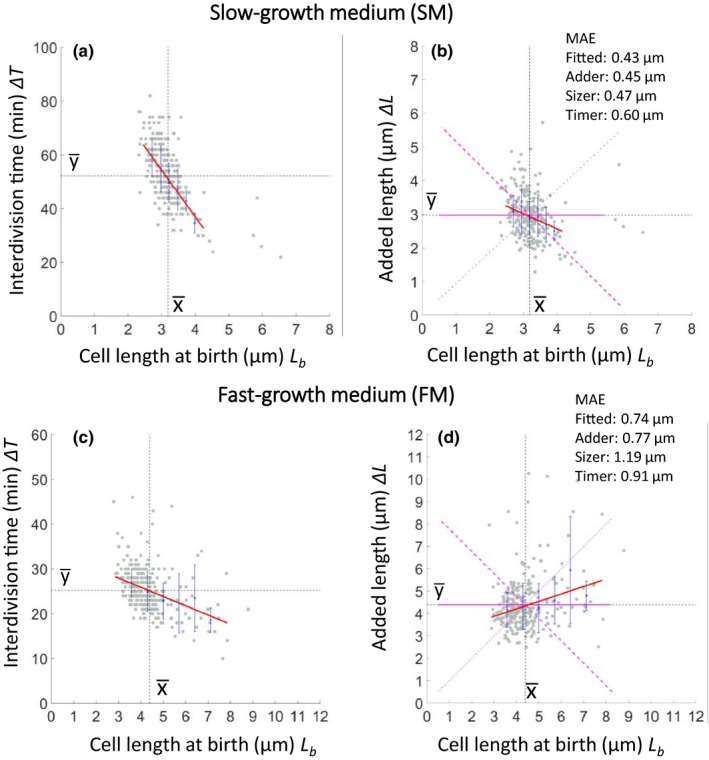
Cell length control in SM and FM. Dependence of interdivision time Δ*T* (a, c) and added length Δ*L* (b, d) on cell length at birth *L*_b_ in SM (a, b) or FM (c, d). Gray dots show each single‐cell data point, and blue crosses and lines show the average of binned data and errors (standard deviation), respectively. Red lines show the linear trend line fits to the single‐cell data. Black dots are guidelines for the mean of *x* $\left(\overset{¯}{x}\right)$ and *y* $\left(\overset{¯}{y}\right)$. The three magenta lines represent expected results for the Adder (solid), Sizer (dashed), and Timer (dotted) models. Numbers in the top right of panels b and d give MAE values for the fitted lines and those of the three models

Figure 3 plots the relationship between cell length at birth (*L*_b_) and elapsed time to next division (interdivision time; Δ*T*), or added length (Δ*L*) for cells grown in FM or SM. In both media, the interdivision time was negatively correlated with the birth length (Figure 3a,c). This was expected, as short new born cells need to grow for a longer period than long newborn cells by any plausible homeostatic mechanism. For added length (Figure 3b,d), the fitted linear regression lines showed slightly different trends. In SM the added length was negatively correlated with length at birth: thus, the cells appear to be able to compensate for being born shorter or longer than average by adjusting the added length. In FM, the trend was slightly different in that long newborn cells tended to overshoot the required growth increment, whereas short cells grew less than expected. Residual analysis was used to test whether the data could be explained by Adder, Sizer or Timer models. The Mean Absolute Error (MAE) values were calculated for the fitted line (red) and for lines (magenta) predicted by the Adder (solid), Sizer (dashed), or Timer (dotted) models. Interestingly, as shown in the top corners of the panels, both Adder and Sizer models accounted for the data in SM almost as well as the fitted lines. However, in FM, the MAE for the Adder model was similar to that of the fitted line but Sizer gave a substantially greater MAE, excluding this as a mechanism for growth homeostasis at the faster growth rate. Timer gave a poor fit to the data under both conditions, as anticipated.

### 3.4 Rate of correction of length fluctuation is influenced by growth rate

**Figure 4:**
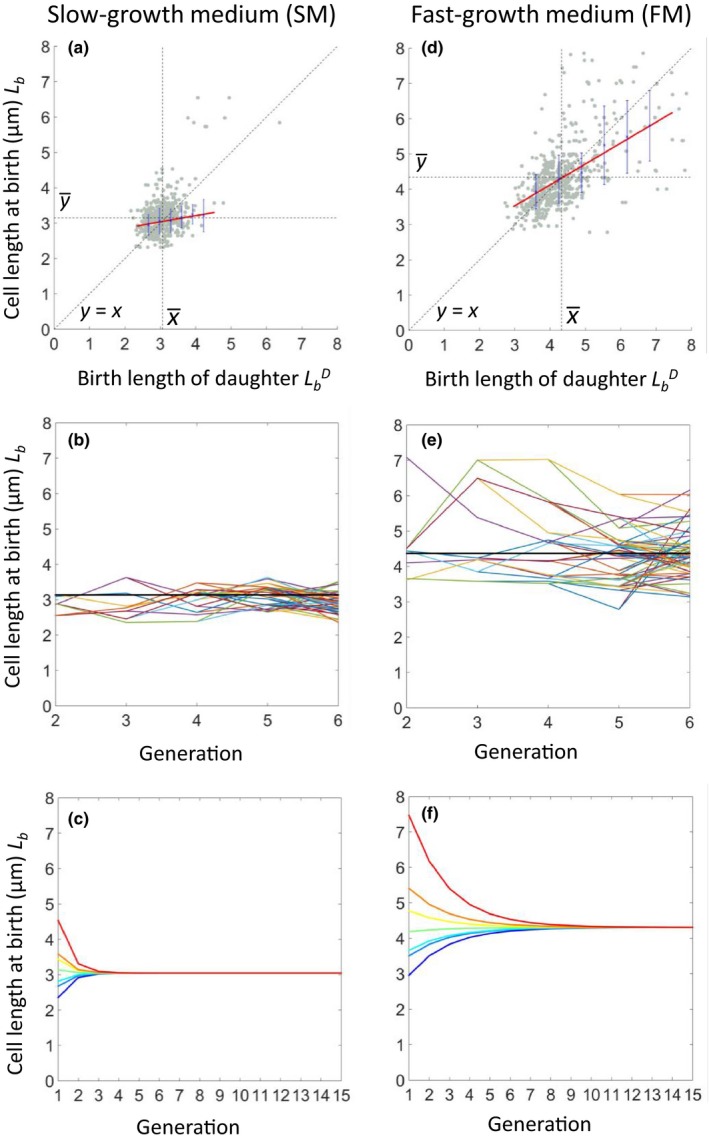
Homeostasis and size convergence in SM and FM. Dependence of (a) birth length of daughter cells over birth length of mother cells in SM. Gray dots show each single‐cell data point, and the blue crosses and lines show the average of binned data and errors (standard deviation), respectively. Red lines show the linear trend line fits to the single‐cell data. Black dots are guidelines for the mean of *x* $\left(\overset{¯}{x}\right)$ and *y* $\left(\overset{¯}{y}\right)$, and *y* = *x*. (b) Exemplar single‐cell traces are shown for cell lineages that were successfully followed for six sequential generations. Different colors show data for different single‐cells, and color lines split when the cells divided. (c) Cell size convergence was simulated by the linear trend line of cell length at birth. Data for the first generation was from the single‐cell data, and color lines are simulation data that show size convergence over 15 generations. (d–f) are the same as (a–c) except that growth was in FM.

One striking feature of the recent time‐lapse analyses of cell cycle progression lies in the degree of imprecision in the behavior of individual cells. The extent to which cells correct deviations from the notional average cell size was assessed by plotting daughter cell length at birth $L_{b}^{\text{D}}$ against that of their parent cell *L*_b_ (Figure 4a,d). (Note that a small number of points were excluded by truncation of the *X*‐axis in Figure 4d.) The results were consistent with the conclusions of the length increment plots. For SM, the low slope of the fitted line, and clustering of points close to the average cell length on both axes show that cells tended to correct fluctuations from average length at the ensuing division. In contrast, in FM, daughter cell length was positively correlated with mother cell length; thus, shorter cells tended to generate shorter daughters and longer cells longer daughters. Panels b and e of Figure 4 show examples of the behavior of individual cell lineages over a series of generations. These plots illustrate how cells do not converge on a preferred average length but oscillate around that length.

Based on the fitted linear trend lines in Figure 4a,d, we modeled the correction of birth length for cells of different starting sizes, assuming perfectly symmetrical divisions. Length correction was achieved in <4 generations for SM (Figure 4c), whereas it required several more generations (about 8) in FM (Figure 4f). One explanation for the difference between the two sets of data would be that at the higher growth rate (in FM) there is insufficient time for cells to carry out the complete set of sequential events needed for accurate regulation of division timing. Nevertheless, the slow rate of correction of cell length in FM would be consistent with an Adder‐like mechanism.

### 3.5 Imprecision in mid‐cell division placement

The requirement for homeostatic mechanisms such as Adder or Sizer implies the existence of sources of variation in cell size. As shown in Figure 3, an important source of this variation is probably imprecision in division timing – the cell is longer or shorter than its “preferred” size (determined by Sizer or Adder effects) at division. However, in principle, it can also arise by variation in placement of the division septum. Some organisms, such as *Caulobacter*, have an intrinsically asymmetric division process (Campos et al., 2014) but the extent to which division in organisms such as *B. subtilis* could be offset from mid‐cell, has not received much attention, in part because time‐lapse imaging is required to capture the moment of division.

**Figure 5:**
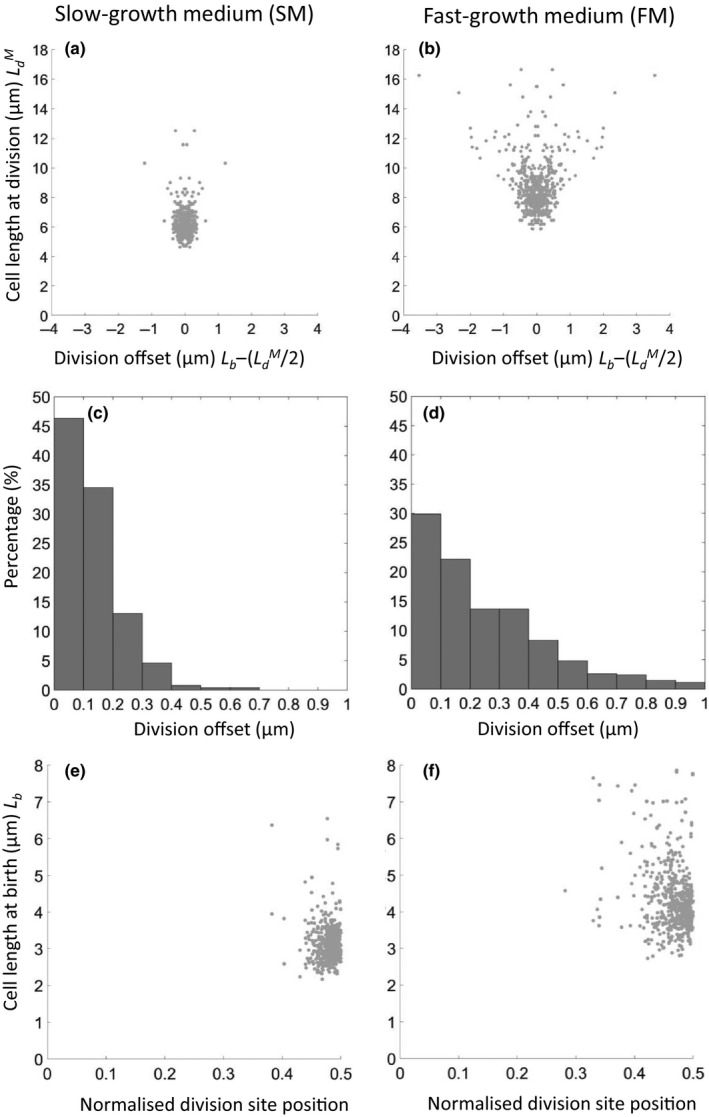
Relative contributions of division length and division offset to variation in birth length. (a, b) Relationship between cell length at division and placement of the division septum (division offset). (c, d) Frequency histograms for binned data on division site offset from mid‐cell. (e, f) relationship between cell length at birth and normalized division site positioning – distance from the division site to nearest cell pole expressed as a fraction of the overall length of the dividing cell. (a, c, e) SM; (b, d, f) FM

**Figure A3:**
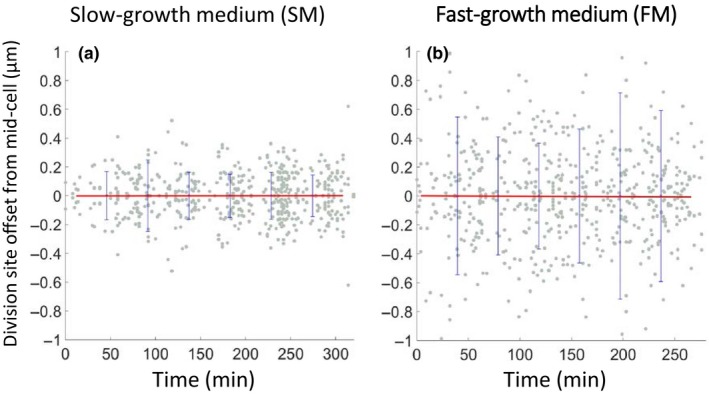
Division site positioning over an extended period of growth. Plot of displacement of division site positioning from mid‐cell during long time course experiments in SM (A) and FM (B). The red line shows the mean, and blue bars the errors (standard deviation) in the binned data [Correction added on 5 July 2019 after first online publication: Figure A3 has been corrected]

We reanalyzed the data from the time‐lapse experiments shown in Figures 2, 3, 4 to compare the relative contributions of variations in division length and division offset to the birth size distribution. Figure 5a,b show plots of cell length at division ($L_{d}^{M}$) against division offset, defined as actual cell length at birth (*L*_b_) minus the expected length if the cell division occurred precisely at mid‐cell ($L_{d}^{M}$/2), for the two media conditions. At both growth rates off‐center division appeared almost as likely in shorter as in longer cells, except that the unusually long cells in FM almost always divided well off center. Relative contributions of the length and offset factors to birth length variation were estimated by comparison of their *SD* values. In SM, length and offset values were 0.94 and 0.17 µm, respectively, indicating that off central division contributed about 15% of the variation in cell size at birth. In FM, the contribution was greater: *SD* values of 1.77 and 0.51 µm, respectively, giving a contribution of 22%. Appendix Figure A3 (Appendix 1) shows that the frequency of asymmetric division did not change appreciably during the 6‐hr time course in the microfluidic channels.

The histograms in Figure 5c,d plot the frequency with which division was placed at different distances from mid‐cell (ignoring, for now, the length of the dividing cell). Taking an arbitrary cutoff of 200 nm as the boundary between central and offset division (based on the approximate level of resolution in our image processing), it appeared that in both SM and FM a substantial proportion of divisions (20% and 50%, respectively) were detectably off center. Thus, by both measures, imprecision in division site placement appears to make a small but significant contribution to cell length variability.

We noted that Migocki, Freeman, Wake, and Harry (2002) had previously reported a high degree of precision in positioning of the FtsZ ring – effectively the key precursor structure to cell division. To compare our data with that of Migocki et al. we replotted division site placement in terms of distance to the nearest cell pole as a fraction of total cell length (i.e., length of the parent cell at division) (Figure 5e,f). The *SD* values for these plots were 0.026 for SM and 0.047 for FM, in the same range as described for placement of the FtsZ ring by Migocki et al. (2002), 0.030. It should be noted that Migocki et al. plotted FtsZ ring position in all cells of the population, including relatively short cells far from being ready for division. Ultimately, direct comparison of FtsZ ring positioning and division site placement in time lapse would be needed to clarify how tightly division site placement is connected to FtsZ ring positioning.

### 3.6 Chromosome termination is not tightly connected to the division cycle

**Figure 6:**
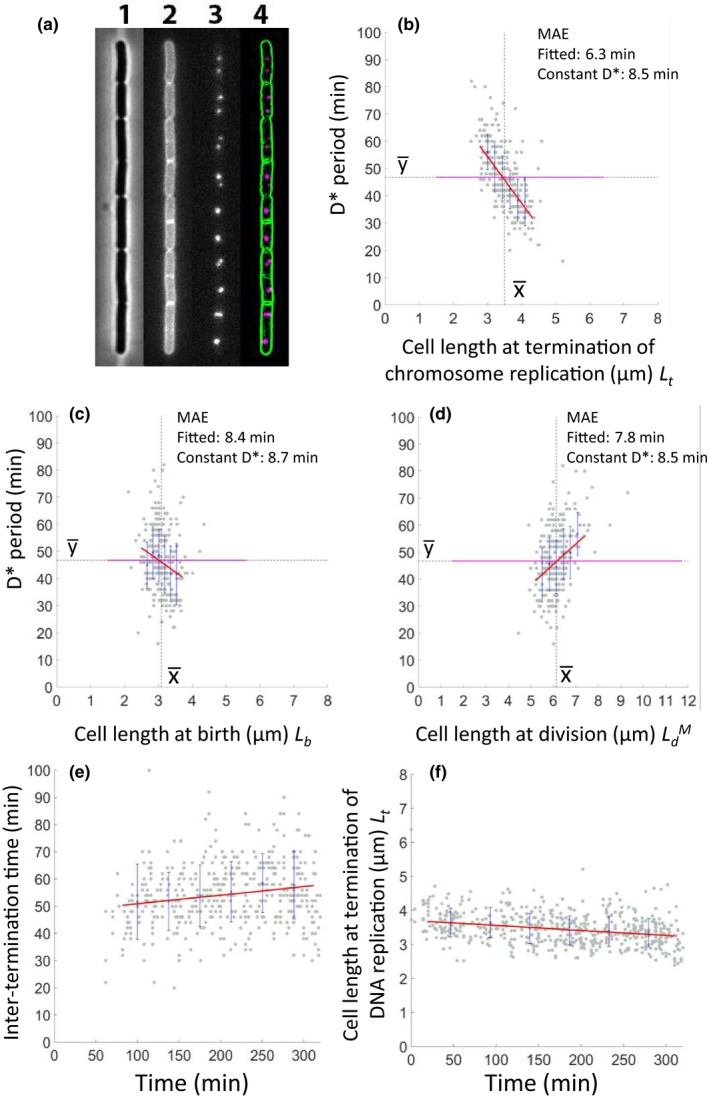
Analysis of the timing of termination of replication and its relationship to cell length. (a) Still images from the time‐lapse series, showing phase‐contrast image (1), GFP (membrane; 2), mCherry (chromosome termini; 3) and afterimage processing (4). (b) Relationship between cell length at termination versus elapsed time to the subsequent division (D*). (c) Relationship between cell length at birth (*L*_b_) and the D* period. (d) Relationship between cell length at division ($L_{\text{b}}^{\text{M}}$) and the D* period. (e,f) Change of Inter‐termination time (e) and change of cell length at termination of DNA replication (f) over the course of the experiment. A *Bacillus subtilis* strain with both the terminus and membrane fluorescent markers (sL099) was grown in SM in agarose‐based microfluidic channels at 32°C for about 6 hr through six generations (*n* = 556). Gray dots show each single‐cell data point, and the blue crosses and lines show the average of binned data and errors (standard deviation), respectively. Red lines show the linear trend lines fitted to the single‐cell data. Black dots and the magenta line are guidelines for the mean of *x* $\left(\overset{¯}{x}\right)$ and *y* $\left(\overset{¯}{y}\right)$ – the latter corresponding to the average D* period. MAE values are shown at the top right of panels b–d

Traditional models of cell division timing have postulated a connection to the termination of chromosome replication. According to the classical Cooper–Helmstetter model (Cooper & Helmstetter, 1968) (Figure 1), division should follow replication termination after a constant period, D (or D* here). Previous population studies of *B. subtilis* were consistent with this model (Sharpe et al., 1998). However, virtually all of the evidence for this coupling has been based on population studies. To test for coupling of termination and division in individual cells, we took the WALP23‐mGFP strain and introduced a *tetO*/mCherry‐TetR system that would label a site close to the terminus of chromosome replication, similar to constructs used to measure terminus position previously (Bogush, Xenopoulos, & Piggot, 2007; Lemon, KURTSER, I., & GROSSMAN, A.D., 2001; Teleman, Graumann, Lin, Grossman, & Losick, 1998; Webb et al., 1997). Figure 6a shows that this enabled us to simultaneously measure both cell length and terminus number in cells growing in the microfluidic system. Under the slow growth conditions used (SM) most cells contained one or two fluorescent terminus foci, representing states before and after termination of a round of replication, respectively. As shown in Figure 6e,f and Movie A3 (Appendix 2) the wild‐type strain with the terminus and membrane fluorescent markers showed only minor changes in growth parameters in the microfluidic device over many generations.

Measurements of several cell growth parameters relative to the timing of duplication of terminus foci are shown in Figure 6b–d. It should be noted that we cannot exclude the possibility of a delay between terminus replication and focal separation. Nevertheless, we found that, in contrast to expectation of a fixed D* period, this parameter showed a strong inverse correlation with the cell length at termination (Figure 6b). The MAE value for the fitted line was much lower than that of the line assuming a fixed D* period. In other words, in longer cells the D* period was proportionately reduced, whereas in shorter cells it was extended. In contrast, cell length at preceding birth, which occurred only a short while before termination of the replication round (average cell length 3.0 vs. 3.5 µm), was not a good predictor of the D* period (MAE values of fitted line and average D* line were similar). The cell length at subsequent division – corresponding to the end of the D* period was actually inversely correlated with the D* value, although again the MAE values of fitted line and D* line were not very different (<10% between MAE values). Nevertheless, this trend would mean that cells that divided later than on average tended to have a longer D* period and vice versa for shorter cells. Thus, completion of chromosome replication does not seem to play a significant role in cell length homeostasis under the slow growth conditions tested. A similar result was recently reported for *E. coli*, using SeqA as a reporter for initiation, progression, and termination of chromosome replication (Adiciptaningrum, Osella, Moolman, Lagomarsino, & Tans, 2015).

## 4 CONCLUSIONS

We have revisited the cell cycle of *B. subtilis* using a microfluidic system and semiautomated time‐lapse imaging, using fluorescent makers for cell division and chromosome termination. The results are generally in line with previous population‐based studies. In light of the recent emphasis on time‐lapse imaging and single‐cell analysis, we can draw several important conclusions. First, our results provide general support for the Adder model for cell size homeostasis, although the relatively low overall precision of division makes it difficult to exclude alternative models. Given that cell cycle progression must be highly important to cells, it would seem to us to be surprising if they did not use a plethora of overlapping regulatory systems to control the decision to divide, and thus not to fit rigorously to any simple growth law. Also, it seems likely that some mechanisms will only come into play in cells that have strayed, for whatever reason, outside of the “normal” range. Second, we find that vegetative *B. subtilis* cells divide off‐center at an appreciable frequency, and that this contributes to the overall variability in cell size. Finally, and probably our most surprising finding, we found little connection between the timing of chromosome termination and subsequent division. Since the D or D* periods, measured at the population level, have historically been shown to be relatively constant across a range of growth rates in both *E. coli* and *B. subtilis*, we expected that this would be reflected in single‐cell analysis. Perhaps the lack of connection is not so surprising, given that the D* period is relatively long (most of the cell cycle) in *B. subtilis*. To conclude, it seems that much remains to be learned about the timing and localization of the bacterial division machinery.

## CONFLICT OF INTERESTS

The authors declare no conflict of interest.

## AUTHOR CONTRIBUTIONS

S.L. performed all the experiments and most of the data analysis. J.E., L.J.W., and S.L. designed the study and wrote the paper.

## ETHICS STATEMENT

None required.

## Data Availability

All data are provided in full in the results section and the Appendices.

## APPENDIX 1

**Table A1:** Summary of measured cell parameters (average values from single‐cell data) [Correction added on 5 July 2019 after first online publication: Interdivision time added for Strain sL099]

Strain	Growth medium	Birth length	Interdivision time	Added length	D* period	Termination length
sL105	SM	3.13 µm ± 0.50	52 min ± 11	2.97 µm ± 0.67		
sL105	FM	4.36 µm ± 1.02	25 min ± 5	4.39 µm ± 1.22		
sL099	SM	3.10 µm ± 0.36	56 min ± 11	3.05 µm ± 0.62	47 min ± 12	3.46 µm ± 0.48

**Table A2:** Bacterial strains and plasmids

Strain/Plasmid	Genotype/description	Source/reference
Strains		
168CA	*trpC2*	Laboratory stock
sL099	168CA *amyE::spc P_xyl_(M9R)‐walp23‐mgfp(A206K)* *dacC::tetO240(171ᴼ) (cat), ycgO::P_ftsW_ tetR‐mCherry (phleo)*	This study
sL105	168CA *amyE::spc P_xyl_(M9R)‐walp23‐mgfp(A206K)*	This study
Plasmids		
pWX510	*bla ycgO::P_ftsW_ tetR‐mCherry (phleo)*	Wang et al. (2014)
pL015	*bla amyE’::spc P_xyl_‐walp23‐mgfp (A206K) ‘amyE*	pSG1154::WALP23‐mGFP of Scheinpflug et al. (2017)
pL062	*bla amyE’::spc P_xyl_(M9R)‐walp23‐mgfp (A206K) ‘amyE*	This study
pCRW10	*bla dacC::tetO240(171ᴼ) (cat)*	C.R. Willis, unpublished
